# Gene Expression Patterns of Oxidative Phosphorylation Complex I Subunits Are Organized in Clusters

**DOI:** 10.1371/journal.pone.0009985

**Published:** 2010-04-01

**Authors:** Yael Garbian, Ofer Ovadia, Sarah Dadon, Dan Mishmar

**Affiliations:** 1 Department of Life Sciences, Ben-Gurion University of the Negev, Beer-Sheva, Israel; 2 National Institute of Biotechnology (NIBN), Ben-Gurion University of the Negev, Beer-Sheva, Israel; Deutsches Krebsforschungszentrum, Germany

## Abstract

After the radiation of eukaryotes, the NUO operon, controlling the transcription of the NADH dehydrogenase complex of the oxidative phosphorylation system (OXPHOS complex I), was broken down and genes encoding this protein complex were dispersed across the nuclear genome. Seven genes, however, were retained in the genome of the mitochondrion, the ancient symbiote of eukaryotes. This division, in combination with the three-fold increase in subunit number from bacteria (N = ∼14) to man (N = 45), renders the transcription regulation of OXPHOS complex I a challenge. Recently bioinformatics analysis of the promoter regions of all OXPHOS genes in mammals supported patterns of co-regulation, suggesting that natural selection favored a mechanism facilitating the transcriptional regulatory control of genes encoding subunits of these large protein complexes. Here, using real time PCR of mitochondrial (mtDNA)- and nuclear DNA (nDNA)-encoded transcripts in a panel of 13 different human tissues, we show that the expression pattern of OXPHOS complex I genes is regulated in several clusters. Firstly, all mtDNA-encoded complex I subunits (N = 7) share a similar expression pattern, distinct from all tested nDNA-encoded subunits (N = 10). Secondly, two sub-clusters of nDNA-encoded transcripts with significantly different expression patterns were observed. Thirdly, the expression patterns of two nDNA-encoded genes, NDUFA4 and NDUFA5, notably diverged from the rest of the nDNA-encoded subunits, suggesting a certain degree of tissue specificity. Finally, the expression pattern of the mtDNA-encoded ND4L gene diverged from the rest of the tested mtDNA-encoded transcripts that are regulated by the same promoter, consistent with post-transcriptional regulation. These findings suggest, for the first time, that the regulation of complex I subunits expression in humans is complex rather than reflecting global co-regulation.

## Introduction

From the time the process of endosymbiosis occurred, mitochondria lost most of their genes to the eukaryotic host genome, retaining only a small circular genome of their own [1], [2], [3]. This extra-nuclear genome, along with its bacterial-like translation machinery and mixed bacterial/phage-like replication and transcription mechanisms mark the mitochondrion as a prokaryotic island embedded within a eukaryotic environment [4], [5]. The bacterial origin of the mitochondrion is clearly reflected by the 37 mitochondrial DNA (mtDNA)-encoded genes, which are transcribed in two polycistrones regulated by the heavy and light strand promoters (excluding the bidirectional promoter in birds) [4], [6]. Thirteen of these genes encode protein subunits of the oxidative phosphorylation (OXPHOS) machinery, which are known to closely interact with nuclear DNA (nDNA)-encoded subunits within four of the five OXPHOS complexes (complexes I, III, IV and V). Two major issues emerge from the nuclear-mitochondrial interactions within the OXPHOS system. Firstly, the mutation rate of the coding mtDNA is higher by an order of magnitude than that of most coding nDNA, thus enforcing tight co-evolution between mtDNA and nDNA-encoded subunits of the OXPHOS mechanism [7], [8]. Secondly, the OXPHOS subunits are not only encoded by two independent genomes in eukarya, i.e. the mtDNA and nDNA, but are further dispersed in different nDNA chromosomes. Such dispersal dramatically challenged the co-regulatory mechanism that used to govern the transcription of these subunits before the radiation of eukaryotes, namely a single operon probably homologous to the NUO operon in bacteria [9]. Is it possible that genes encoding protein subunits comprising the eukaryotic OXPHOS complexes retained some patterns of co-regulation, despite their division between the mtDNA and nDNA?

Genome-wide analysis of high-quality human core promoter sequences revealed, that most promoters enriched with YY1 elements were associated with mitochondrial genes [10]. Moreover, regions harboring promoters of nDNA-encoded OXPHOS genes were enriched with certain transcription factor recognition motifs [11], [12]. Analysis of microarray transcriptional patterns of various OXPHOS genes in humans suggested clustering of transcripts encoding elements of the same OXPHOS complex [11], thus, conceivably facilitating co-regulation of OXPHOS genes' expression.

Here, we analyzed the expression pattern of 17 complex I subunits comprising all mtDNA and representative nDNA-encoded subunits in 13 human adult and fetal tissues. Although we found some support to previously argued co-regulation of complex I genes we found clear sub-clustering of expression patterns. We also found that the expression patterns of mtDNA- and nDNA-encoded subunits diverge and that certain nDNA-encoded subunits diverge from the general nDNA-encoded complex I subunits pattern of transcription. These results shed new light on the complex regulation mode of the steady-state levels of complex I subunits transcripts.

## Results

We aimed at assessing possible co-regulation of genes encoding complex I subunits at the transcripts level. To this end, we analyzed the steady-state transcript levels of seventeen different complex I subunits by real time PCR in 13 different tissues (referred to here as ‘expression patterns’), including 9 adult and 4 fetal tissues. We normalized the transcript levels of expression to that of glyceraldehyde 3-phosphate dehydrogenase (GAPDH) as a reference gene (see methods section). The studied complex I transcripts included ten nDNA-encoded and all seven mtDNA-encoded subunits (Table 1). Of these nine subunits are human orthologues of bacterial proteins comprising the set of ‘core subunits’ (i.e. all 7 mtDNA and two of the nDNA-encoded subunits, NDUFV1 and NDUFS2). The remaining eight tested subunits belong to the group of ‘supernumerary’ nDNA-encoded subunits which were gradually recruited to complex I after the radiation of eukaryotes [13]. The chosen subunits are localized in different compartments of complex I, with 13 being embedded within the hydrophobic arm (comprising 7 mtDNA- and 6 nDNA-encoded subunits) and four being localized in the matrix (hydrophilic) arm (Table 1). Apart from the seventeen complex I subunits, we included in the framework of our expression pattern analysis the beta-actin gene representing a non-mitochondrial housekeeping gene. Similar to the studied complex I subunits, the expression pattern of beta actin was also normalized to GAPDH. Because our analysis reflects the expression pattern relative to GAPDH rather than the absolute transcription level no conclusions could be drawn regarding the **absolute** levels of transcripts of the tested genes in each of the tissues. Instead, we focused on comparing the **relative** transcript levels and patterns among the genes, across all tissue samples.

**Table 1:** The tested complex I subunits, their genome affiliation (mtDNA or nuclear DNA), and their location in complex I.

Gene Name	Genome	Recruitment during evolution	Location in complex I
ND1	mtDNA	Core subunit	Hydrophobic arm
ND2	mtDNA	Core subunit	Hydrophobic arm
ND3	mtDNA	Core subunit	Hydrophobic arm
ND4	mtDNA	Core subunit	Hydrophobic arm
ND5	mtDNA	Core subunit	Hydrophobic arm
ND6	mtDNA	Core subunit	Hydrophobic arm
ND4L	mtDNA	Core subunit	Hydrophobic arm
NDUFS2	nDNA	Core subunit	Hydrophilic arm
NDUFV1	nDNA	Core subunit	Hydrophilic arm
NDUFA1	nDNA	Supernumerary (*eukarya*)	Hydrophobic arm
NDUFA4	nDNA	Supernumerary (*Insecta*)	Hydrophobic arm
NDUFA5	nDNA	Supernumerary (*eukarya*)	Hydrophilic arm
NDUFA10	nDNA	Supernumerary (*Metazoa*)	Hydrophobic arm
NDUFA12	nDNA	Supernumerary (*eukarya*)	Hydrophilic arm
NDUFB10	nDNA	Supernumerary (*eukarya*)	Hydrophobic arm
NDUFB11	nDNA	Supernumerary (*eukarya*)	Hydrophobic arm
NDUFC2	nDNA	Supernumerary (*Metazoa*)	Hydrophobic arm

### Relative levels of mtDNA-encoded gene transcripts are higher than those of nDNA-encoded subunits

**Figure 1:**
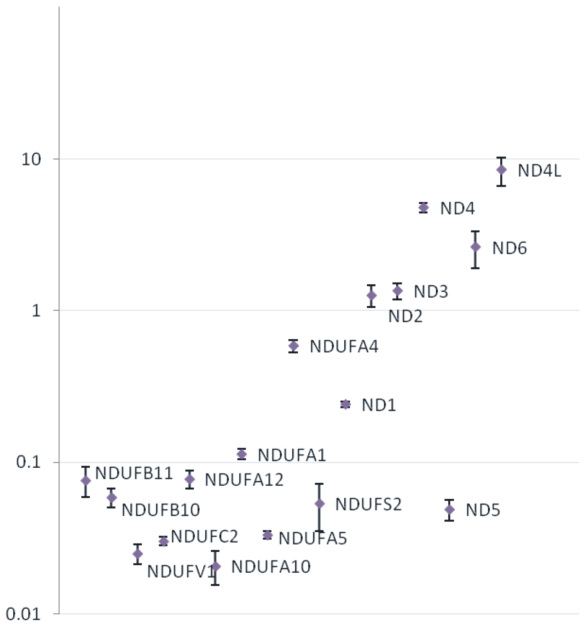
Relative expression levels of the different subunits in a representative tissue on a logarithmic scale. This figure demonstrates the expression pattern of all tested complex I transcripts in brain medulla as a representative tissue. The error bars were calculated using three independent replication experiments. Y axis indicates in a logarithmic scale the relative transcripts levels measured by Real time PCR and normalized by that of the reference gene (GAPDH).

We first assessed whether differences in the relative transcript levels of the subunits could be noted among the tested tissues. A comparison of the GAPDH-normalized levels of the transcripts in each tissue revealed differences in the transcript levels of the genes encoding the various subunits of the complex, as previously reported [14] (the expression pattern in a representative tissue is demonstrated in Figure 1). In general, transcript levels of most mtDNA-encoded subunits seemed higher than that of the nDNA-encoded subunits. However, there were notable differences in the transcript level among the subunits. Specifically, in most tissues ND4L showed a relatively higher transcript level than most of the other mtDNA-encoded subunits by more than one order of magnitude. ND5 exhibited the lowest relative transcript level as compared to most of the mtDNA-encoded subunits, and was expressed at a level similar to the nDNA-encoded subunits. Similarly, the relative transcript levels of nDNA-encoded subunits differed from one another by up to one order of magnitude. NDUFA4 showed an order of magnitude higher transcript level as compared to most of the nDNA-encoded subunits and was expressed to a degree similar to the relative transcript level of the mtDNA-encoded ND1 subunit.

### Transcripts of complex I subunits are expressed in clusters

**Figure 2:**
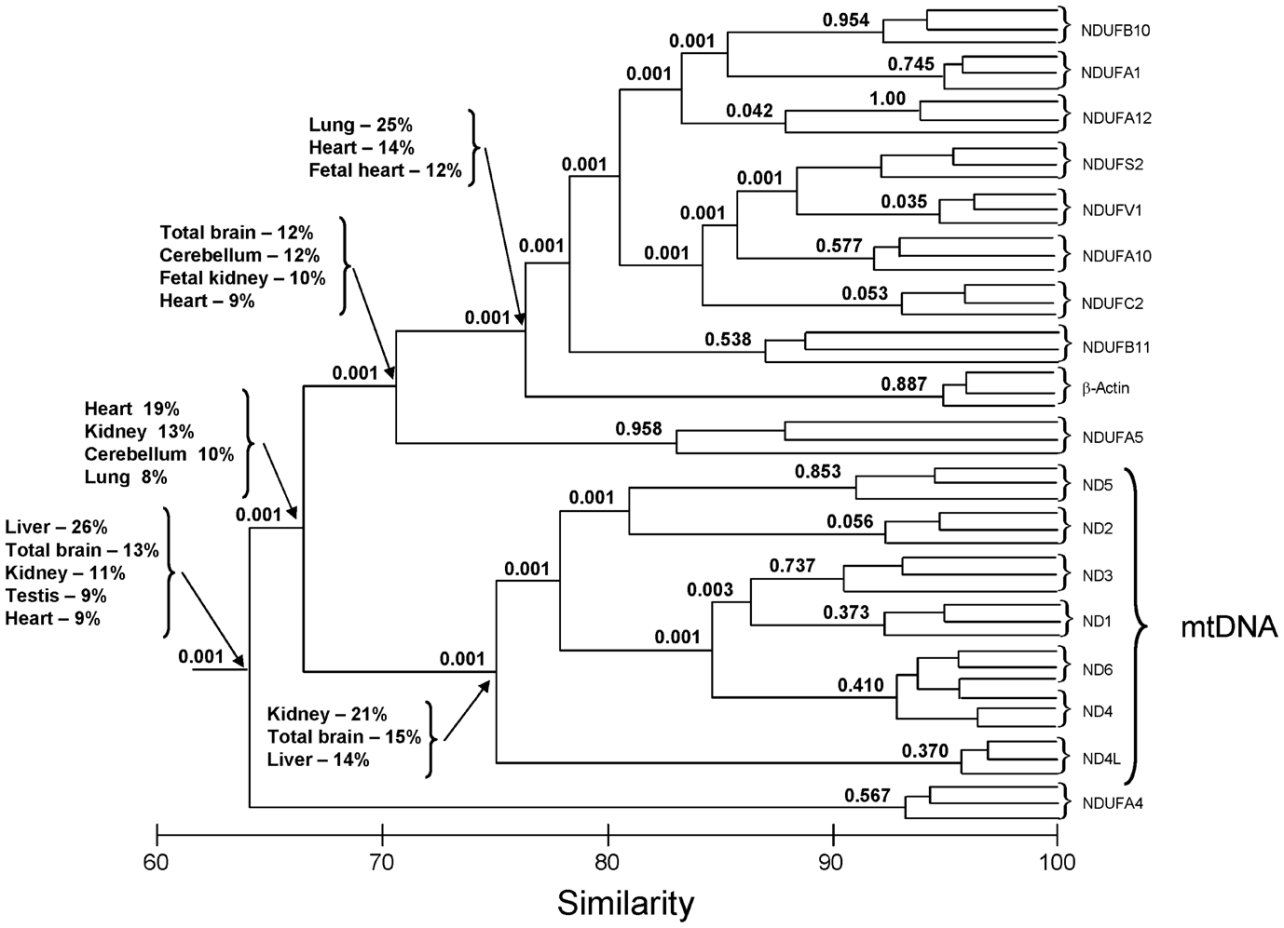
Cluster analysis of the tissue expression pattern in complex I genes. Numbers above branches represent p-values reflecting the significance of the clustering (see methods section). Tissues most contributing to the branching order are mentioned below each branch. Relative expression patterns of mtDNA and nDNA-encoded complex I subunits were normalized to a reference gene (GAPDH) and hence do not represent absolute quantification of transcripts levels in the tested tissues but rather a relative expression pattern within each tissue.

To perform an overall comparative analysis of the expression patterns of the various subunits, we carried out cluster analysis considering the GAPDH-corrected transcript levels of each subunit in all 13 tested tissues. Such an analysis was aimed at assessing similarities and differences in the patterns of expression of all the subunits in a tree-based manner. This analysis suggested that the expression pattern of mtDNA-encoded subunits was significantly distinct from all of the nDNA-encoded subunits (Figure 2). Among the nDNA-encoded genes two significantly distinct clusters of expression patterns could be identified with the first including NDUFS2, NDUFV1, NDUFA10, NDUFC2 and NDUFB11 and the second including NDUFB10, NDUFA1 and NDUFA12. It is worth noting, that although the analysis identified a general clustered expression patterns of most nDNA-encoded subunits, these subunits also significantly clustered with beta-actin thus questioning global co-regulation of complex I genes. NDUFA4, and to a lesser extent, NDUFA5, showed (each) different expression patterns than did the other tested nDNA- and mtDNA-encoded subunits. When inspecting the cluster of expression of mtDNA-encoded subunits, ND4L presented a significantly distinct pattern, which diverged from those of the other mtDNA-encoded subunits.

When examining the tissues most contributing to the dissimilarities in expression patterns (Figure 2), heart and kidney contributed most to the differences noted between the mtDNA-encoded subunits cluster and the nDNA-encoded subunits. NDUFA5 pattern of expression differed from the nDNA-encoded subunits expression pattern mostly in total brain and in the cerebellum. The expression pattern of NDUFA4 differed from both the expression patterns of nDNA- and mtDNA-encoded subunits mostly in liver, total brain and kidney. ND4L expression differed from that of the other mtDNA-encoded subunits especially in kidney and total brain. The tissues presenting expression patterns of the nDNA-encoded subunits most different from that of beta-actin were lung and heart. Interestingly, the tissues most contributing to the differences in expression between mtDNA-encoded subunits and beta-actin were also the lung and the heart (not shown).

## Discussion

It is logical to assume the existence of a mechanism that governs co-regulation of the subunits of OXPHOS complex I so as to allow proper complex assembly and function. This hypothesis is supported by the facts that genes encoding proteins that collaborate often tend to be co-regulated at the mRNA level [15] and that the transcription levels of many OXPHOS genes are altered together in metabolic disorders, such as type 2 diabetes mellitus [16]. To test for such co-regulation, we investigated the steady state levels of transcripts encoding 17 complex I subunits (i.e., all seven mtDNA-encoded and ten representative nDNA-encoded subunits) in a variety of human adult and fetal tissues. Our findings revealed the existence of at least two clusters of expression among complex I subunits, one composed of all mtDNA-encoded subunits and a second composed of most of the tested nDNA-encoded subunits. Deeper investigation revealed that heart and kidney are the major contributors to the divergence in mtDNA- and nDNA-encoded subunits expression. During the course of our work, a microarray-based analysis revealed evidence for co-transcription of OXPHOS genes, showing the preference of subunits of each OXPHOS complex to cluster separately [11]. This view gained partial support from two of our evidences. Firstly, although the expression pattern of beta-actin branched closer to the nDNA than to the mtDNA-encoded genes, most of the tested nDNA-encoded complex I subunits (NDUFB10, NDUFA1, NDUFA12, NDUFS2, NDUFV1, NDUFA10, NDUFC2 and NDUFB11) formed a significantly distinct cluster that was separated from beta-actin. Secondly, when examining the tissues most contributing to the differences between beta-actin versus the nDNA-encoded and mtDNA-encoded subunits transcript levels, both nDNA- and mtDNA-encoded subunits were higher than beta-actin in heart and notably lower than beta-actin in lung (data not shown). However, the existence of distinct sub-clusters of expression patterns among nDNA-encoded genes supports co-regulation among sub-groups of complex I subunits. This finding, in conjunction with the clustering of most nDNA-encoded genes with beta actin, suggests complex regulatory scheme rather than global co-regulation of all nDNA-encoded subunits of the complex. Nevertheless, to further assess modes of co-regulation among complex I subunits, the full set of 45 subunits should be tested.

The diverging expression pattern of NDUFA4 (and to a lesser extent, NDUFA5) from the rest of the tested nDNA-encoded subunits attracted our attention. The tissues contributing mostly to these differences were liver and total brain in the case of NDUFA4, and cerebellum and total brain (with cerebellum probably most contributing to the total brain difference) in the case of NDUFA5. This observation could result from a different mode of regulation of these subunits, at least in the mentioned tissues. However, since our observed relative expression patterns reflect the steady-state level of the transcripts, we could not distinguish whether the observed differences were due to transcriptional and/or post transcriptional regulatory mechanisms. Nevertheless, the sharp divergence of NDUFA4 expression pattern raises the possibility that this subunit serves functions in addition to its role in complex I.

In agreement with previous reports, the transcript levels of mtDNA-encoded subunits were generally higher than those of the nDNA-encoded subunits (Figure 1 and [14]). Although all mtDNA-encoded subunits shared a similar expression pattern, the ND5 transcript exhibited a lower transcription level than did the rest of the mtDNA-encoded subunits by one order of magnitude in most tested tissues (Figure 1). In contrast, ND4L exhibited notably higher transcription levels from the rest of the mtDNA-encoded subunits by one order of magnitude in most of the tested tissues (Figure 1). In addition, the expression pattern of ND4L significantly differed from that of the rest of the mtDNA-encoded subunits, with kidney and total brain being the tissues mostly contributing to this difference (Figure 2). When considering that mtDNA-encoded subunits are transcribed in a polycistronic fashion and are regulated by the same promoter (excluding ND6) [17], these observations support the existence of post-transcriptional regulatory mechanisms and/or differences in mRNA stability among mtDNA-encoded genes. Post-transcriptional regulation was previously observed for mtDNA-encoded tRNA genes, suggesting that such regulation is not restricted to protein-coding mtDNA genes [18]. In summary, the expression pattern analysis of complex I subunits was in line with co-transcription of mtDNA-encoded subunits and provided evidence for co-regulation of groups of nDNA-encoded subunits with two tested subunits (NDUFA4 and NDUFA5) that diverged from the rest of the complex. Hence, for the first time, we support the possibility that the transcriptional regulation of complex I genes is organized in sub-clusters.

### Conclusions

In our study, we provide a detailed assessment of the steady state transcript expression patterns of both nDNA and mtDNA-encoded subunits of OXPHOS complex I. We showed that the expression of mtDNA-encoded subunits clustered separately from that of nDNA-encoded subunits. Considering that most nDNA-encoded subunits (i.e., NDUFB11, NDUFS2, NDUFA1, NDUFA10, NDUFA12, NDUFB10, NDUFC2 and NDUFV1) formed a distinct expression cluster, the concept of co-regulation is partially supported. However, the distinct sub-clusters of groups of nDNA-encoded subunits in combination with the divergent expression pattern of the nDNA-encoded subunit NDUFA4 provided first clues for a complex regulatory scheme of complex I subunits. The distinct expression pattern of NDUFA4 mostly due to differences in its transcription in liver, total brain and kidney, suggests possible tissue-specific function either within or outside of complex I activity. Taken together our analysis suggests that the transcriptional regulation of complex I subunits is organized in clusters, paving the path towards investigating possible associations of complex I regulation with tissue-specific energetic requirements.

## Materials and Methods

### RNA and cDNA extraction and purification

Total cDNA was produced from commercially available human total RNA (Ambion FirstChoice) from 13 different tissues (Ambion FirstChoice and Clontech: kidney, cerebellum, brain medulla, total brain, skeletal muscle, heart, liver, lung, testis, fetal kidney, fetal heart, fetal brain, fetal liver) using an iScript cDNA Synthesis kit (BIO-RAD), according to the manufacturer's protocol. Each of these RNAs were reported to include pooled samples of at least 3 unrelated individuals, thus partially correcting for individual differences in expression levels. The products were transferred to -20°C for storage. cDNA was used for subsequent real time PCR amplification of the following gene transcripts: NDUFA1, NDUFA4, NDUFA5, NDUFA10, NDUFA12, NDUFB10, NDUFB11, NDUFS2, NDUFC2, NDUFV1, GAPDH, beta-actin and the mtDNA-encoded subunits ND1-ND6 and ND4L. The PCR products were transferred to −20°C for storage.

### Real Time PCR

Relative quantification of the steady-state transcript levels of the nDNA- and mtDNA-encoded subunits of complex I was performed using Real time PCR. 100–300 ng of cDNA from each of 13 different normal human tissues served as templates for separate Real time PCR amplifications (PCR reaction volume of 20 µl containing 1 X Absolute SYBR Green ROX Mix, Thermo), and 100 nM of each specific primer (Table 2). The following PCR protocol was used in a Stratagene MX3000P Real Time PCR machine: 15 seconds at 95°C followed by 40 cycles including denaturation for 30 seconds at 95°C, annealing for 1 minute at 60°C and extension for 30 seconds at 72°C. Each of the experiments was performed in duplicate tubes, and was repeated three times in different days. Glyceraldehyde 3-phosphate dehydrogenase (GAPDH) was used as a reference gene, in each experiment. For correct quantification, the amount of cDNA used for the amplification of GAPDH was adjusted according to the amount of each of the amplified genes. For example, genes amplified using 100 ng cDNA per well, were normalized to the amount of GAPDH amplified in the same amount of cDNA. To control for DNA contamination in the reaction mix, control tubes lacking DNA templates (NTC) were included in duplicate with the relevant set of primers in each experiment. NTC tubes were also included in triplicate in each standard curve experiment. Standard curves were generated in triplicates for each primer sets to assess the efficiency of the reaction with one of the cDNAs mentioned above.

**Table 2:** Real Time PCR Primers employed in this study.

Gene Name	Primer sequences	Product size (bp)	Amount of cDNA (ng/well)
NDUFA1 (MWFE)	F:ATGTGGTTCGAGATTCTCC R:GCAACCCTTTTTTCCTTGC	116 bp	200 ng/well
NDUFA4 (MLRQ)	F:CAGAGCCCTGGAACAAACTGGG R:GACCTTCATTCTAAAGCAGCG	137 bp	250 ng/well
NDUFA5 (B13)	F:GAGAAGCTGGCTATGGTTAAAGCG R:CCACTAATGGCTCCCATAGTTTCC	154 bp	300 ng/well
NDUFA10 (42 kd)	F: CACCTGCGATTACTGGTTCAG R:GCAGCTCTCTGAACTGATGTA	130 bp	250 ng/well
NDUFA12 (DAP-13)	F:ACATTCTGGGATGTGGATGG R:CTAGTGGTAGAATAAGGTAC	156 bp	250 ng/well
NDUFB10 (PDSW)	F:TAGAGCGGCAGCACGCAAAG R:CTGACAGGCTTTGAGCCGATC	188 bp	200 ng/well
NDUFB11 (ESSS)	F:GGAAAGCGGCCCCCAGAACCGAC R:CCACGCTCTTGGACACCCTGTGC	231 bp	100 ng/well
NDUFC2 (B14.5b)	F:GGTTTGCATCGCCAGCTTC R:CAGGAAAATCCTCTGGATG	137 bp	200 ng/well
NDUFS2 (49KD)	F:ACCCAAGCAAAGAAACAGCC R:AATGAGCTTCTCAGTGCCTC	214 bp	200 ng/well
NDUFV1 (51kd)	F:TGAGACGGTGCTGATGGACTTC R:AGGCGGGCGATGGCTTTC	113 bp	250 ng/well
ND1	3439H:CTACTACAACCCTTCGCTGAC 3655L:GGATTGAGTAAACGGCTAGGC	216 bp	100 ng/well
ND2	4892H:CATATACCAAATCTCTCCCTC 5166L:GTGCGAGATAGTAGTAGGGTC	274 bp	100 ng/well
ND3	10166F:TTACGAGTGCGGCTTCGACC 10355R:ACTCATAGGCCAGACTTAGG	189 bp	100 ng/well
ND4	11269H:CTAGGCTCACTAAACATTCTA 11455L:CCTAGTTTTAAGAGTACTGCG	186 bp	100 ng/well
ND4L	10528H:TAGTATATCGCTCACACCTC 10726L:GTAGTCTAGGCCATATGTG	198 bp	100 ng/well
ND5	13627H:TCGAATAATTCTTCTCACCC 13725L:TAGTAATGAGAAATCCTGCG	98 bp	100 ng/well
ND6	14258L:GGATCCTCCCGAATCAAC 14359H:GTAGGATTGGTGCTGTGG	119 bp	100 ng/well
GAPDH	F:GAAGGTGAAGGTCGGAGTC R:GAAGATGGTGATGGGATTTC	200 bp	100 ng/well
β-actin	F:CGCGAGAAGATGACCCAGAT R:TCACCGGAGTCCATCACGAT	126 bp	100 ng/well

Real time PCR Mx3000P software was used to determine the amplification cycle in which product accumulation was above the threshold cycle values (Ct). Real time PCR Ct values were analyzed using the 2^-ddCt^ method [19]. The mean of Ct duplicate tubes for a given gene and tissue was normalized to the mean Ct value of the reference gene (GAPDH) from the same tissue in each experiment, as follows: To reveal similarities or differences in the expression pattern among the 17 tested complex I subunits in a panel of 13 different tissues, the 2^−dCt^ of each gene product from a given tissue was calculated as a portion of the sum of all 2^−dCt^ values of the same transcript in all tested tissues.

### Analysis of similarity between the expression patterns of complex I subunits

A total of 51 expression profiles were obtained from the Real-Time PCR analyses (17 subunits ×3 replicates = 51 expression profiles). Each of the profiles consist of 13 values (corresponding to the 13 tested tissues) that were standardized by total 2^−dCt^ corresponding to the different tissues examined. Using the PRIMER v6 software (PRIMER-E Ltd, Plymouth, UK), we generated a similarity matrix comprising Bray-Curtis similarity coefficients (Eq. 7.24 in [20]) of all the possible pairwise transcription profile comparisons. Each of these coefficients represents the resemblance between two expression profiles. Next, to find the ‘natural groups’ of the 51 expression profiles, namely when expression profiles within a group are more similar to each other than to profiles in different groups, we used hierarchical agglomerative clustering [20]. This was followed by a series of ‘similarity profile’ (SIMPROF) permutation tests, looking for statistically significant evidence for genuine clusters in the generated tree diagram (dendrogram). Specifically, tests were performed, at every node of the computed dendrogram, such that the group being sub-divided had significant (P<0.05) internal structure. Identical dendrogram was obtained when Euclidian distance was used instead of Bray-Curtis similarity coefficients (data not shown). Finally, to asses the contribution of each of the 13 tissues examined to each of the significantly detected expression clusters we used the ‘similarity percentages’ routine (SIMPER), which decomposes average Bray-Curtis dissimilarities into percentage contributions. It is notable, as expected, that the three independent replicates of each of the 17 subunits were clustered together, supporting the quality of the experiments.
